# Supplementary material for: Antioxidant Effect of a Dietary Supplement Containing Fermentative S-Acetyl-Glutathione and Silybin in Dogs with Liver Disease
Source: Vet Sci. 2023 Feb 8;10(2):131. doi: 10.3390/vetsci10020131 (PMC9966841; doi:10.3390/vetsci10020131)
Supplement: Supplementary file 1 [file vetsci-10-00131-s001.zip › vetsci-2132004-supplementary.pdf]

Supplementary file.

Table S1. Biochemical parameters of both groups (control -CTR and treated -TRT group) at the baseline (T0). Total proteins (TP), albumins (ALB), glucose (GLU), alanine transaminases (ALT) alanine aminotransferases (AST), alkaline phosphatases (ALP), Gamma-glutamyl transferase (GGT), bilirubin (BIL), triglycerides (TRI), c- reactive protein (PCR). Minimum and maximum values of the tested parameters are indicated in parentesis.

Dog	Group	BIL (0-0,7 mg/dL)	ALT (0-40 UI/L)	AST (0-40 UI/L)	GGT (2-8 UI/L)	TP (5,4-7,5 g/dL)	ALB (2,3-3,9 g/dL)	GLU (67-132 mg/dL)	PCR (0-1,5 mg/L)	TRI (23-110 mg/dl)	ALP (20-150 UI/L)
D1	TRT	2,79	120	219,95	34,23	6,5	3,7	123,1	1	55,7	156,33
D2	TRT	2,96	131,48	225,32	13,8	8,2	3,2	70,7	1,1	116,8	356,08
D3	TRT	1,23	293,46	348,13	33,22	6,4	2,8	80,1	0,6	156	165,25
D4	TRT	1,71	384,43	306,54	32	7,9	3,7	114,7	0,6	129,2	391,27
D5	TRT	3,75	217,5	205,84	38,16	7,2	2,8	71	0,2	112,4	230,37
D6	TRT	3,6	252,91	240,87	14,66	6,9	3	108	1,2	120,3	150,63
D7	TRT	3,41	501,4	439,55	22,73	7,5	2,8	129,5	0,8	167,1	331,14
D8	TRT	2,27	560,02	331,47	21,61	5,7	3,3	123	1,5	130,6	159,27
D9	TRT	1,38	418,02	364,79	34,39	6,7	2,3	86	0,3	199,3	392,08
D10	TRT	1,23	331,54	357,51	25,19	5,4	2,6	121,2	1,4	150,4	279,69
D11	TRT	0,81	311,03	177,62	30,95	7,4	3	84,7	1,4	161,2	231,63
D12	TRT	2,13	438,31	408,25	13,25	6,5	3,4	128,8	1,5	97,8	344,25
D13	CTR	3,45	117,84	139,42	8,76	6	2,6	108,1	1,3	170,7	197,45
D14	CTR	1,51	492,84	482,86	9,16	6,3	3,8	71,9	1,5	57	198,45
D15	CTR	2,52	526,23	399,08	22,53	7,2	3	77,9	1,4	6,1	199,45
D16	CTR	3,23	337,38	385,94	23,66	6,8	3,6	67,9	1	75,7	200,45
D17	CTR	3,8	253,85	392,96	7,44	5,9	2,8	129,8	1,2	219,2	201,45
D18	CTR	3,07	161,69	483,09	21,06	6,1	3,9	75,1	1,1	186,2	202,45

D19	CTR	2,35	428,63	434,6	32,4	5,3	3,7	107,5	1,5	159,8	203,45
D20	CTR	3,51	295,49	359,54	39,01	7,5	2,6	83,6	1,5	130,7	204,45
D21	CTR	0,78	198,49	85,17	33,89	5,7	3,9	110,1	0,8	83,5	205,45
D22	CTR	1,57	173,16	132,78	13,28	6,2	2,6	128,9	0,8	92,5	206,45
D23	CTR	2,81	378,71	136,56	10,41	6,8	3	89,3	1,3	190	207,45
D24	CTR	3,54	268,73	112,34	9,08	6,3	3,2	96,7	0,7	163,8	208,45
